# Achieving high strength and high ductility in magnesium alloy using hard-plate rolling (HPR) process

**DOI:** 10.1038/srep17100

**Published:** 2015-11-25

**Authors:** Hui–Yuan Wang, Zhao–Peng Yu, Lei Zhang, Chun–Guo Liu, Min Zha, Cheng Wang, Qi–Chuan Jiang

**Affiliations:** 1 Key Laboratory of Automobile Materials of Ministry of Education & School of Materials Science and Engineering, Nanling Campus, Jilin University, No. 5988 Renmin Street, Changchun 130025, P. R. China

## Abstract

Magnesium alloys are highly desirable for a wide range of lightweight structural components. However, rolling Mg alloys can be difficult due to their poor plasticity, and the strong texture yielded from rolling often results in poor plate forming ability, which limits their further engineering applications. Here we report a new hard-plate rolling (HPR) route which achieves a large reduction during a single rolling pass. The Mg-9Al-1Zn (AZ91) plates processed by HPR consist of coarse grains of 30–60 μm, exhibiting a typical basal texture, fine grains of 1–5 μm and ultrafine (sub) grains of 200–500 nm, both of the latter two having a weakened texture. More importantly, the HPR was efficient in gaining a simultaneous high strength and uniform ductility, i.e., ~371 MPa and ~23%, respectively. The superior properties should be mainly attributed to the cooperation effect of the multimodal grain structure and weakened texture, where the former facilitates a strong work hardening while the latter promotes the basal slip. The HPR methodology is facile and effective, and can avoid plate cracking that is prone to occur during conventional rolling processes. This strategy is applicable to hard-to-deform materials like Mg alloys, and thus has a promising prospect for industrial application.

Magnesium (Mg) alloys are being increasingly evaluated for lightweight structural materials due to the compelling need for energy-efficient engineering systems^1^,^2^,^3^,^4^. In the recent decade, many studies have been focused on ultrafine-grained Mg alloys, with the application of severe plastic deformation (SPD) techniques, including equal-channel angular pressing (ECAP)^5^,^6^, differential speed rolling (DSR)^7^ and accumulative roll bonding (ARB)^8^,^9^, etc. The ultrafine grains really induce excellent strengthening in Mg alloys; however, they generally cause premature fractures and result in low ductility at room temperature. For instance, an ECAP processed Mg-9Al-1Zn alloy^5^ exhibited an ultimate tensile strength as high as 410 MPa, but its total elongation was just 8.5%. This drawback in ductility limits the widespread engineering applications of Mg alloys.

Improving the ductility at room temperature has been a vital requirement for ultrafine-grained Mg alloys. One approach is to activate basal slip activity by developing a weakened texture. Nevertheless, this approach often reduces the strength below the level of the starting material owing to texture softening effect^10^,^11^,^12^,^13^. Dispersing nanosized particles in ultrafine grain interiors was proved to be an efficient way to simultaneously increase the strength and ductility^14^,^15^,^16^. Although an obvious strengthening was attained, the prolonged elongation remains inadequate. A major reason for this is the limited deformation systems inside the fine/ultrafine and highly textured grains. Considére criterion^17^,^18^,^19^ suggests that ultrafine-grained material is prone to plastic instability unless larger grains of appropriate sizes and volume fractions are present. Actually, bimodal grain structures have been successfully prepared in some ultrafine-grained materials, such as Cu^19^, Ni^20^,^21^, Al^22^ and TiAl^23^, and consequently leading to an excellent toughening.

The deformation mechanism of Mg alloys depends on a combination of grain size and crystallographic orientation. For this reason, to increase the ductility of Mg alloy sheets, it not only needs to generate a structure containing a mixture of large grains and fine grains but also requires weakening the texture of the sheets. However, due to the fact that Mg alloys usually suffer from poor plasticity and are prone to oxidation, it is hard to achieve a bimodal grain structure and weakened texture simultaneously using traditional preparation method. Therefore, it is in urgent need that we develop a new preparing technique for Mg alloys.

Conventional Mg alloys rolling techniques usually resort to multi-pass rolling with a relatively small reduction per pass, which is beneficial to achieve a uniform deformation, homogeneous grain structure and strong texture, but does not help in harvesting high plastic sheets. In contrast, a single-pass large-reduction rolling process results in uneven deformation, and thus has a probability to achieve bimodal or multimodal grain structures and weakened texture. However, due to the extremely large shear forces in the rolling direction (RD), Mg alloy sheets are very prone to cracking, and thus this rolling technique can hardly be used for production. Aiming at decreasing the shear force in the RD, we proposed a hard-plate rolling (HPR) technique (see Fig. 1), i.e. sandwiching the sample between two hard-plates and then rolling the sandwich structure, which partially transforms the shear stress along RD to compressive stress along normal direction (ND), skillfully solving the cracking problem occurred during the rolling process.

The purpose of this study is to develop a new rolling technique, which is applicable to hard-to-deform materials, e.g., Mg alloys. The multimodal grain structure and texture achieved were investigated, and tensile properties of the AZ91 Mg alloy fabricated by the HPR process were also tested. Our results demonstrated that the HPR can largely reduce the occurrence of edge cracks during rolling, increasing the reduction in a single pass and improving rolling efficiency. Importantly, the tensile strength and ductility of the Mg alloys achieved can be improved simultaneously. It is believed that our strategy is readily applicable to Mg alloys and other hard-to-deform materials, and has a promising prospect for industrial application.

## Results

### The initial microstructure and texture

Field emission scanning electron microscopy (FESEM) observations (Fig. 2(a,b)) show that the initial material of AZ91 alloy after extrusion and pre-heat treatment at 688 K exhibited a uniform grain structure, where most of the Mg_17_Al_12_ particles distributed along grain boundaries whereas a few in grain interiors. Moreover, the electron backscattered electron (EBSD) and pole figure results (Fig. 2(c,d)) reveals that in addition to a strong basal texture, there is also a weakened texture component that rotated from the basal plane towards the extrusion direction (ED).

### A multimodal grain structure

The AZ91 plate used in this study was fabricated by the HPR process. FESEM observations (Fig. 3a) indicate that the resulting microstructure exhibits a typical mixed grain structure, which is mainly composed of coarse grains of 30 ~ 60 μm (Fig. 3a,c) and fine grains that are smaller than 5 μm (Fig. 3b). The bulk volume fraction of coarse grains is about 40% (Fig. 3d). The macrotexture measured by X-ray diffraction (XRD) (Fig. 3e) indicates that the sheet exhibits a basal texture with most of the c-axes aligned parallel to the ND. Note that the spreading of basal poles is relatively large, especially along the TD. To analyze the orientation of coarse and fine grains, the sheet is examined via EBSD (Fig. 4). The result obtained from RD-TD plane shows that although the basal plane of coarse grains is parallel to the rolling plane (Fig. 4b), that of fine grains forms certain angles with the rolling plane (Fig. 4c). Statistical analysis of the microtexture indicates that a weakened texture features in fine grains (Max = 8.2), whereas coarse grains exhibit a strong basal texture (Max = 24.5) (Fig. 4b,c). To further study the texture feature of the mixed grain structure, the RD-ND plane of the sample was also characterized by EBSD (Fig. 5). We can see clearly that the orientations of coarse grains are mainly 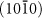 and 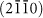 prism planes, and those of fine grains varies significantly, even including some ones having (0001) basal plane. Interestingly, the morphologies of coarse grains are elongated along RD, which implies that most of the coarse grains exhibit American football shapes rather than spherical. As can be seen from the misorientation profile (Fig. 5b), a long range misorientation of ~16˚ exists across the grain within a distance of ~40 μm, while several subgrain boundaries with misorientation angles of 2 ~ 5˚ exist in the grain interiors (Fig. 5b,c). It means that subgrain boundaries have already formed in coarse grains during the HPR process. However, due to the difficulty in using scanning step size smaller than 500 nm during EBSD mapping of the deformation structure of Mg alloys, more detailed characterization of the submicron structures in areas dominated by fine/ultrafine grains has to be done by transmission electron microscopy (TEM).

TEM observations (Fig. 6a) indicate that the microstructure obtained here also involves a large number of ultrafine (sub) grains with a size ranging from 200 to 500 nm. The grain boundaries (GBs) of most of the ultrafine grains are not sharp and no GB phase is detected (see hollow arrows in Fig. 6a). Besides, a large quantity of second-phase Mg_17_Al_12_ particles^14^ are precipitated out in the sheet and randomly dispersed in grains, whose shape is primarily spherical and the size is estimated to be less than 100 nm (see solid arrows in Fig. 6b).

### High strength and high ductility

Tensile stress-strain curves (Fig. 7a) show that AZ91 alloy sheet prepared via HPR has a high strength and strong work hardening ability: the 0.2% offset yield strength (σ_0.2_) is about 221 MPa, the tensile strength (σ_b_) is ~371 MPa, and the work hardening exponent (n) is ~0.27 (Table 1). Moreover, the sheet has a high tensile ductility at room temperature, with an elongation-to-failure (δ_f_) of ~26%. In contrast, the typical strength of as-cast AZ91 alloys is ~250 MPa, and the total elongation is 5–10% (Fig. 7b). Also, both the strength and elongation of the HPRed Mg sheets are superior to those of AZ91 sheets prepared by conventional extrusion, where the latter usually possesses a strength <~350 MPa and elongation <~15%. As shown in Fig. 7b, there is a trade-off between the strength and ductility for all AZ91 alloys. Both yield strength and the ultimate strength of the sample processed by the current HPRed AZ91 alloy clearly fall outside of the reported ordinary strength- ductility trade-off.

Uniform elongation is suggested to be a more appropriate measure for the ductility of fine-grained materials^24^. Interestingly, the uniform elongation of the HPRed Mg sheets is approximately equal to its δ_f_, i.e., ~23% (Table 1). In fact, the measured uniform ductility for the HPRed Mg sheets is almost twice as large as those reported for conventionally deformed AZ91 sheets, e.g., the uniform elongation of the wrought alloys is merely around 10–15% (Fig. 7b). Although the strength of AZ91 sheet can be greatly improved by SPD processes like ECAP, ARB and DSR, which is usually at the expense of uniform ductility, i.e., only of a few percent (Fig. 7b). Therefore, it is clear that the present AZ91 sheet processed by HPR possesses a superior combination of high strength and high uniform ductility.

Uniform elongation is mainly controlled by work hardening rate, θ^25^,^26^. The work-hardening rate, 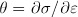, where 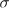 is the true stress and 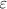 is true strain, was calculated according to the true stress-strain curves, and the θ−σ and θ−ε plots are shown in Fig. 7c. Initially, θ values are quite high and decrease rapidly with increasing strain, but the declining rate of θ becomes obviously slower and keeps almost constant at later tension stages. Moreover, θ always keeps at higher values than σ until the fracture point, i.e., a strain level of ~23%. This is in agreement with the observation of a significant uniform elongation combined with no sudden necking appearing in the stress-strain curve (Fig. 7a).

## Discussion

**Figure 1:**
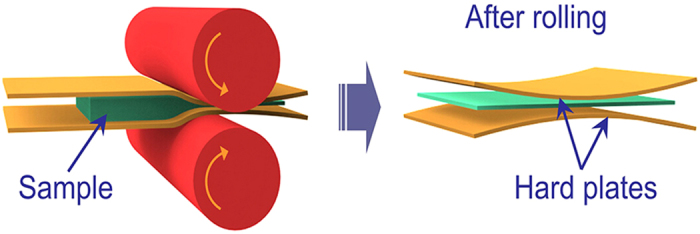
Schematic illustration showing the process of hard-plate rolling (HPR).

**Figure 2:**
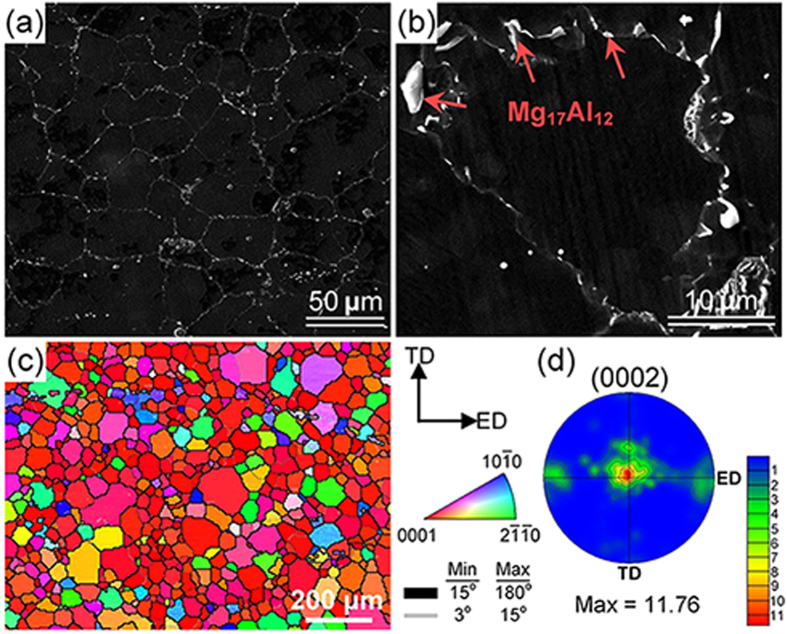
(**a**) Typical FESEM micrograph of the initial material of AZ91 sheet (after extrusion and pre-heat treatment at 688 K); (**b**) High magnification of FESEM showing most of the Mg_17_Al_12_ particles distributing along grain boundaries whereas a few in grain interiors; (**c**) Typical FESEM-OIM maps taken in the ED-TD plane of the initial AZ91 sheet; (**d**) The microtextures obtained from (**c**).

**Figure 3:**
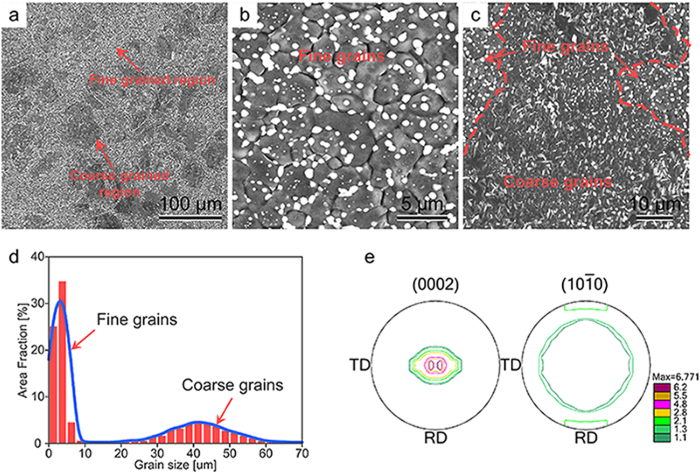
(**a–c**) FESEM micrographs of the HPRed AZ91 sheet, where (**b**,**c**) are the details of the marked regions in (**a**); (**d**) grain size statistics showing the bimodal grain size distribution in the HPRed AZ91 sheet; (**e**) pole figures showing the texture of the HPRed AZ91 sheet.

**Figure 4:**
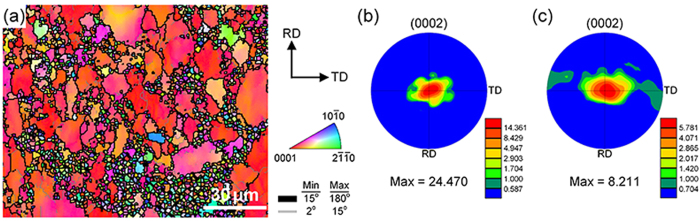
(**a**) Typical FESEM-OIM maps taken in the RD-TD plane of the HPRed AZ91 sheet; (**b,c**) are the microtextures of the coarse and fine grains, respectively.

AZ91 alloy is one of the most widely used commercial high strength Mg alloys, and is mainly used for die casting production. Although the grains of AZ91 alloys can be greatly refined by SPD, the simultaneous improvement of strength and ductility remains a challenge (Fig. 7b). In some cases, the ductility even goes lower. Inspired by the fact that a bimodal grain structure can improve the strength and ductility simultaneously^19^,^20^,^21^,^22^,^23^, we proposed a new HPR route (Fig. 1) which introduces a large reduction during a single rolling pass, and therefore, a desired multimodal grain structure was readily achieved in the AZ91 alloys (Figs 3a, 4a and 6a).

Due to the existence of heated hard-plates between the rolls and Mg sheets, heat dissipation from the sheets was depressed significantly, which thus decreased cracking during rolling efficiently. Moreover, shear stress applied on the sample along RD was partially transformed to pressure stress along ND due to the adding of two hard plates, and consequently inducing a large thickness reduction and improving rolling efficiency. It makes HPR suitable for rolling large-scale alloy sheet, which is difficult to be achieved in traditional SPD processes. Thus, it is believed that HPR has promising prospects for industrial applications.

**Figure 5:**
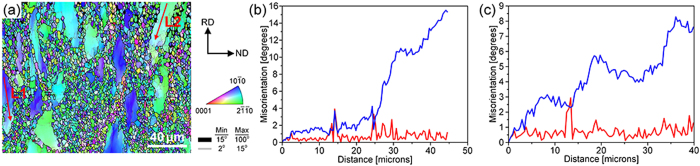
(**a**) Typical FESEM-OIM maps taken in the RD-ND plane of the HPRed AZ91 sheet; misorientation profiles measured along (**b**) line L1 and (**c**) line L2, indicated in Fig. 5a.

It is of great interest and worthy to discuss the underlying mechanisms that control the generation of the multimodal grain structure, i.e., why a large fraction of submicron gains formed and coexisted with micron-sized coarse grains in the HPRed Mg alloy. As for the formation of coarse American-football shape grains (Figs 4a and 5a), the reason is quite obvious, i.e., due to the extension of the grains during HPR process.

The misorientation profiles along lines measured from selected coarse grains that shown in Fig. 5 could be used to interpret the formation mechanism of the fine/ultrafine grains. As can be seen from the misorientaion profile (Fig. 5(b)), a large accumulated misorientation gradient of ~16˚ exists across the grain within a distance of ~45 μm, although the relative misorientations are almost constant and below 2^o^. Secondly, several LAGBs already had formed, e.g., see Fig. 5(b,c), indicating the possibility in growing into HAGBs by further accumulation of dislocations. In fact, such obvious microstructure features associated with continuous dynamic recrystallization (CDRX), i.e., proceeded by a continuous absorption of dislocations in sub boundaries (LAGBs) and eventually resulting in the formation of new grains bounded by HAGBs, were frequently observed in the coarse grains in bimodal grain structures in severely deformed Al alloys^22^. Furthermore, as the newly generated fine/ultrafine (sub) grains were evolved from large misorientation gradients, it is not surprising that even the neighboring fine/ultrafine (sub) grains could have quite different orientations. Also, once the fine/ultrafine (sub) grains formed, they are likely to rotate from their original orientations during further deformation, which further increase the (sub) boundary misorientaitons of neighboring grains. Therefore, the fine/ultrafine (sub) grains formed after the HPR process features more or less random orientations and thus a weakened texture.

**Figure 6:**
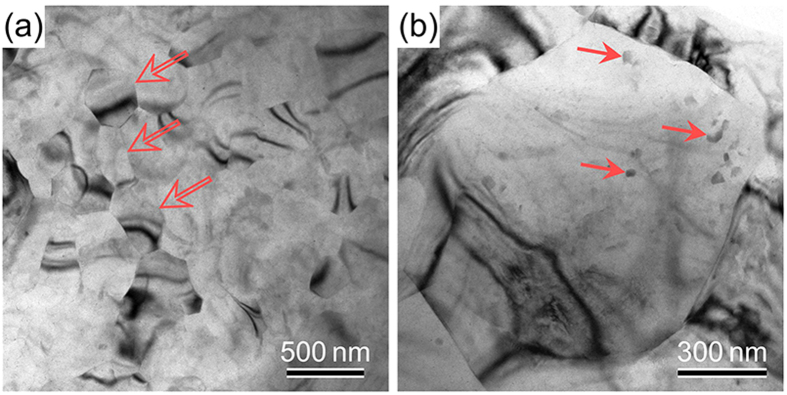
(**a**,**b**) TEM micrographs showing the ultrafine subgrains (indicated by open arrows) and nanosized Mg_17_Al_12_ particles (indicated by solid arrows) in the HPRed AZ91 sheet.

**Figure 7:**
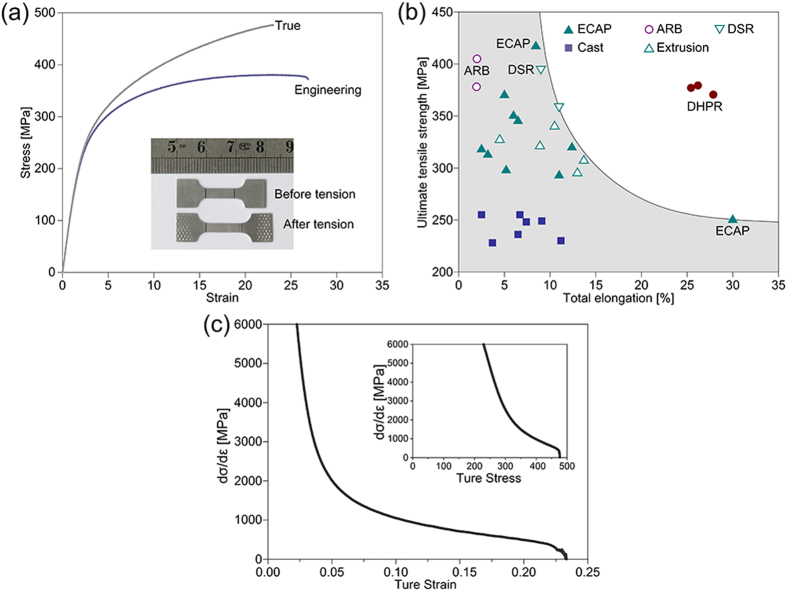
(**a**) Stress-strain curves of the HPRed AZ91 sheet; (**b**) Tensile strength versus total tensile elongation of AZ91 alloys in comparison with available literature data (ECAP^6^,^9^,^24^,^30^; ARB^8^,^31^; DSR^32^; Cast^7^,^14^,^24^,^33^; Extrusion^24^,^34^); (**c**) work-hardening rate as a function of true strain and true stress of the HPRed AZ91 sheet.

**Figure 8:**
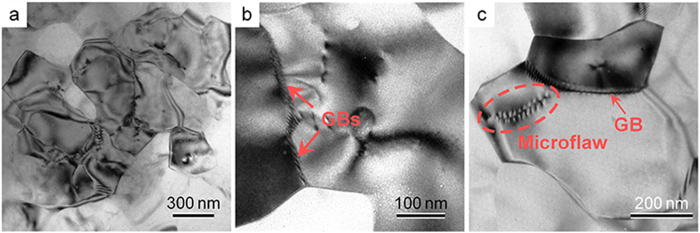
(**a–c**) TEM micrographs showing the confined dislocation pile-ups and microflaw in the HPRed AZ91 sheet after a tensile strain of 20%.

A central question to be addressed in our present study is what is responsible for the simultaneous improvement of strength and ductility of the AZ91 sheet processed by HPR. It is evident from the FESEM (Fig. 3) and TEM (Fig. 6) observations that the increased strength should be mainly attributed to the grain refinement strengthening (i.e. the formation of fine and ultrafine grains) and second-phase strengthening. Such a deduction can be further corroborated by the finding dislocation pileups (micro-flaws) in the ultrafine (sub) grains after tensile test, which was especially obvious at grain boundaries (GBs) (Fig. 8a–c). In addition, the subgrain boundaries formed in the coarse grains (Fig. 5(b,c)) would hinder the dislocation motion and contribute to the strength increment. However, the improvement of ductility was chiefly due to two reasons: i) the fine grains of different orientations (Figs 4 and 5) and with the size less than 5 μm benefit for the activation of basal and non-basal slips^27^, which can largely improve the uniform deformation of Mg alloys, and ii) the existence of coarse grains facilitates twining during tensile deformation, resulting in the increase of the number of slip systems^28^,^29^, i.e. twinning-mediated plasticity.

The high uniform ductility in the present HPRed Mg sheets should be also attributed to the strong work hardening that resulted from the multimodal grain structure. Earlier literature demonstrated that a multimodal grain structure is efficient in maintaining strong work hardening and hence uniform deformation in fine-grained materials, as the large grains are effective in storing dislocations^25^,^26^. Ultrafine and fine grains have limited capacity for dislocation accumulation, so that the initial high work hardening rate fades rapidly as the ultrafine and fine grains saturate with dislocations soon. However, coarse grains have the capability to further accommodate newly formed dislocations, maintaining a moderate work hardening until a much larger deformation.

As a result, it is rather safe to say that a superior combination of high strength and high uniform ductility of AZ91 alloy sheet processed by HRP mainly benefits from the cooperation effect of a multimodal grain structure and weakened texture.

## Conclusions

In the present work, we proposed a new HPR route, where a large reduction can be achieved by merely a single rolling pass. The HPR was demonstrated to be an efficient methodology in facilitating the formation of a multimodal grain structure and weakened texture simultaneously. The HPRed AZ91 plates consisted of coarse grains of 30–60 μm, exhibiting a typical basal texture, and fine grains of 1–5 μm as well as submicron-grains of 200–500 nm, the latter two having a weakened texture. More importantly, a simultaneous high strength and uniform ductility (~371 MPa and ~23%) was gained in the AZ91D alloy developed by HPR. The high strength is mainly attributed to the formation of fine/ultrafine grains and also second-phase strengthening. The high uniform ductility should be primarily due to the fine grains featuring with random orientations that induce basal and non-basal slip as well as coarse grains favoring twinning that coordinate plastic deformation. The strong work hardening that resulted from the multimodal grain structure is also responsible for the high uniform ductility in the present HPRed Mg sheets. The developed HPR process is facile and effective, and can avoid plate cracking which is prone to occur during conventional rolling processes. This strategy is applicable hard-to-deform materials like Mg alloys, and hence has a promising prospect for industrial application.

## Methods

### Materials fabrication

Alloys with composition of Mg-9.20Al-0.65Zn (wt.%) were melted and cast to a rod measuring 95 mm in diameter. After that, the rod was extruded at 663 K to a bar with a cross-section of 40 mm × 5 mm (designated as as-received alloy). Hard-plate rolling (HPR) that added hard plates (made of hardened steel ~50 HRC, thickness of 1 mm) between rolls and sample during rolling, was applied in this study. The hard plates were preheated together with samples. Before HPR processing, the extruded alloys were solid solutionized at 688 K for 20 h and cooled in cold water, to obtain supersaturated solid solutions. The quenched alloy was preheated at 623 K for 10 min and then deformed to 0.75 mm (thickness reduction Δε = 85%) by one pass of HPR. The obtained sheet was held at 623 K for 7 min and rolled again by HPR, to flat and homogenize the alloy. The sheet with a final thickness of 0.71 mm after two passes of HPR is referred to as HPRed sheet. The roll velocity was 12 m/min, and the rolls were not heated. After HPR process, the sheets were annealed at 623 K for 5 min.

### Characterization of mechanical properties and microstructures

For mechanical property measurements, all the samples were cut and polished to a cross-section of 4 mm × 0.7 mm and a gauge length of 10 mm, with axes along the rolling direction (or extrusion direction). Uniaxial tensile tests were conducted on a MTS-810 testing system at a constant strain rate of 1 × 10^–3^ s^–1^ at room temperature. Microstructures of samples were observed by field emission scanning electron microscopy (FESEM) (FEI-XL 30) and transmission electron microscope (TEM) (JEM-2100 F) respectively. The representative FESEM and TEM micrographs displayed in this paper are all based on more than 20 different fields. Quantitative evaluations of grain sizes were statistically analyzed using ~1000 grains in FESEM micrographs and ~1200 particles in TEM micrographs respectively. The macrotextures of the samples were examined by X-ray diffraction on a Rigaku 2500PC X-ray diffractometer with Cu Kα radiation at 40 kV and 150 mA.

To gain reliable electron backscattered electron (EBSD) images, the mechanical polished samples were ion milled for 50–60 min. by using focused ion beam at a gas flow rate of ~0.07 ml/min. In order to decrease the potential damage of the crystal lattice upon exposure of the focused ion beam, a low voltage of 3 V and a high tilt angle of ~80° were applied during ion milling. EBSD studies were carried out in a Zeiss 55VP FEG-SEM equipped with a Nordif EBSD detector. TSL OIM software was used for the analysis of the EBSD images. EBSD characterization was performed with 20 kV acceleration voltage, 22 mm working distance, 70° tilt, and with 0.2–0.5 μm scan steps depending on the magnifications.

## Additional Information

**How to cite this article**: Wang, H.-Y. *et al.* Achieving high strength and high ductility in magnesium alloy using hard-plate rolling (HPR) process. *Sci. Rep.* **5**, 17100; doi: 10.1038/srep17100 (2015).

## Figures and Tables

**Table 1:** Tensile strength (σ_b_), yield strength (σ_0.2_), elongation-to-failure (δ_f_), uniform elongation (δ_p_), strain hardening capacity (*H*_*c*_) and strain hardening exponent (*n*) of AZ91 alloy sheet processed by HPR.

Tensile strength, *σ*_*b*_/MPa	Yield strength, *σ*_0.2_/MPa	Elongation-to-failure, *δ*_*f*_/%	Uniform elongation *δ*_*p*_/%	Hardening capacity, H_c_	Strain hardening exponent, n
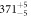	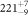	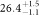	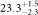	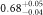	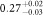
